# Teaching for the future

**DOI:** 10.7554/eLife.05846

**Published:** 2015-01-13

**Authors:** Indira M Raman

**Affiliations:** Department of Neurobiology, Northwestern University, Evanston, United Statesi-raman@northwestern.edu

**Keywords:** living science, grad school, education, careers in science, history of science, scientific publishing

## Abstract

Reading and discussing classic papers can be an effective way of teaching graduate students how to learn the skills they will need for a career in research, as **Indira M Raman** explains.

These days I find myself repeatedly discussing with colleagues the question of how to train graduate students so that they will be well equipped to conduct the research of the future. Not only must we give students a broad-based introduction to the field, the conversation usually goes, but we must also provide them with all the knowledge required of a modern scientist: statistics, computer programming, optics, ethics, and translational science, among others. As a distressing vision of a decade-long graduate program gradually emerges, my colleagues and I end up acknowledging that we cannot teach students everything they must know for their careers, especially in an ever-evolving discipline. We must teach them to learn. So how do we do that?

I suspect the answer is not unique. Perhaps what matters is not so much what we teach, but how we teach it, since nearly any topic may serve as a medium to encourage students to think, to learn, and to think about learning, so that they ultimately develop the skill—and courage—to train themselves. For me, this medium comes through my graduate course on the historical literature, in which we study the landmark papers that provide the basis for ideas that are now considered to be ‘true’. We call it ‘Great Experiments’.

Some scientists wonder why, in this age of information, one would force students to read papers whose technology and terminology are outdated, and whose lasting conclusions are summarized in the first chapters of any textbook, if not on Wikipedia itself. Indeed, most students already know the outcomes of the experiments, and usually their first impression is simply that the old papers are long. Some exceed 50 pages, with laborious descriptions of tubes and transistors, or how many millimeters of saline covered a bit of tissue.

In class, we begin by mentally entering the historical era of the research. What was known before the experiments were performed? What did the authors want to know? What technology was available? What did they build from scratch or assemble from components with other uses? Students think about the design, construction, and use of devices that have been superseded by the elaborate instrumentation of their own labs. The disparity between old and new sparks their curiosity about equipment they currently use. Who invented it? How does it work? Could it be made differently? These questions lead students to confront their own lack of technical knowledge, often by asking somewhat shyly after class where they can learn electronics or get access to a breadboard. One day they might confess apologetically that they only know how to use software-simulated oscilloscopes; the next week they may report with satisfaction their success with a borrowed but real oscilloscope. Some students have even dared to delve into the theoretical sections of user manuals.

Next, we examine the language of the text. What did the authors mean when they used a particular phrase to draw attention to an observation? Remarkably, we discover that unfamiliar phrases refer to ideas that we now take for granted—in fact, because of the results in this very paper. The authors express themselves cautiously, though, and surprisingly, they sometimes describe phenomena with phrasing that now sounds slightly inaccurate. It is a revelation for many students that, when a discovery is made, researchers are forced to articulate ideas that were previously unspoken because they didn't exist. In a sort of intellectual bootstrapping, scientists must fashion words imperfectly to represent new phenomena, or cobble together phrases that pull meanings from other sources to create new concepts. Students start to notice words that they previously ignored or wonder whether a meaning is hidden in an odd-sounding sentence. Language reveals itself as a tool to be handled as precisely as a microscope or an amplifier. ‘Can you take responsibility for every word *you* put on the page?’ Sometimes I ask students this question, and sometimes they ask it of themselves.

Naturally, we delve into the science of the papers as well. Many students are impressed that the original authors were so exact in listing possible errors about interpretations that now seem incontrovertible. The realization that those authors knew—and openly acknowledged—the limits of their revolutionary discoveries offers a contrast with modern papers, which often strike the novice as unassailable. Our class discussions wander into the relationships between new research articles and early work. Does a recent study directly confirm a previously indirect inference? Does it provide extension of an earlier discovery? An exception to a rule? Or does it really overturn a long-held but mistaken idea? As modern scientific literature gains a context, students start to see each published paper as representing a moment in a history that climaxes with them.

Reading the old literature brings up still broader issues about current scientific practice. The question that recurs yearly is: ‘Why don't we write like this anymore?’ Why, the students ask, are articles so much briefer, with incidental observations and detailed alternatives reported only rarely? Why don't we discuss sources of error so extensively? If the culture of science is driven largely by peer review, did scientists themselves choose this style? Or have other forces determined the landscape of science?

Recently, we read a paper in which the same control was performed repeatedly to verify the accuracy of the signals that were recorded. As the experiments progressed, the authors became adept at recognizing and discarding contaminated measurements and could proceed without the verifications. The students identified with this experience, noting that intimacy with their experiments had taught them, too, to detect subtleties that previously seemed invisible. Learning to respond to such details in the data not only accelerates research, they agreed, but it also avoids inaccuracies. With practice, subjectivity evolves into expertise.

As we reached this consensus, it seemed an opportune moment to bring up the present interest in data sharing, the notion that scientists will soon upload all raw data to the cloud for widespread use by others. ‘What will happen,’ I asked, ‘when data are divorced both from their experimental context and from the knowledge and skills of the investigators who gathered them?’ The students' responses were serious and thoughtful, measured and sincere. At least for the moment, I felt confident entrusting the future of science to them.

**Figure 1:**
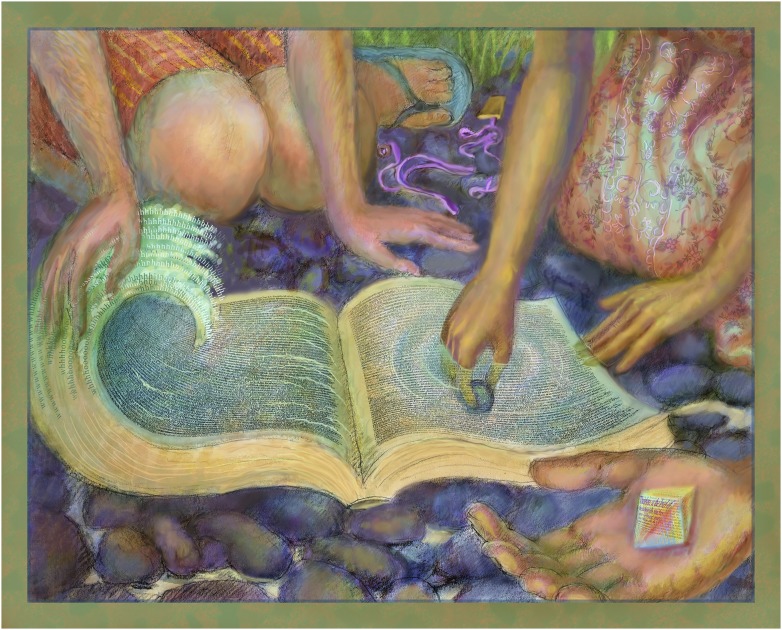
Landmark papers can be used to teach students about many different aspects of modern science.

ILLUSTRATION: BEN MARDER

